# Genome-Wide Association Study Identifies *Nox3* as a Critical Gene for Susceptibility to Noise-Induced Hearing Loss

**DOI:** 10.1371/journal.pgen.1005094

**Published:** 2015-04-16

**Authors:** Joel Lavinsky, Amanda L. Crow, Calvin Pan, Juemei Wang, Ksenia A. Aaron, Maria K. Ho, Qingzhong Li, Pehzman Salehide, Anthony Myint, Maya Monges-Hernadez, Eleazar Eskin, Hooman Allayee, Aldons J. Lusis, Rick A. Friedman

**Affiliations:** 1 Graduate Program in Surgical Sciences, Federal University of Rio Grande do Sul, Porto Alegre, Rio Grande do Sul, Brazil; 2 Department of Otolaryngology, Zilkha Neurogenetic Institute, USC Keck School of Medicine, University of Southern California, Los Angeles, California, United States of America; 3 Department of Preventive Medicine and Institute for Genetic Medicine, USC Keck School of Medicine, University of Southern California, Los Angeles, California, United States of America; 4 Department of Human Genetics, University of California, Los Angeles, Los Angeles, California, United States of America; 5 Department of Computer Science, University of California, Los Angeles, Los Angeles, California, United States of America; 6 Department of Microbiology, Immunology, and Molecular Genetics, University of California, Los Angeles, Los Angeles, California, United States of America; Tel Aviv University, ISRAEL

## Abstract

In the United States, roughly 10% of the population is exposed daily to hazardous levels of noise in the workplace. Twin studies estimate heritability for noise-induced hearing loss (NIHL) of approximately 36%, and strain specific variation in sensitivity has been demonstrated in mice. Based upon the difficulties inherent to the study of NIHL in humans, we have turned to the study of this complex trait in mice. We exposed 5 week-old mice from the Hybrid Mouse Diversity Panel (HMDP) to a 10 kHz octave band noise at 108 dB for 2 hours and assessed the permanent threshold shift 2 weeks post exposure using frequency specific stimuli. These data were then used in a genome-wide association study (GWAS) using the Efficient Mixed Model Analysis (EMMA) to control for population structure. In this manuscript we describe our GWAS, with an emphasis on a significant peak for susceptibility to NIHL on chromosome 17 within a haplotype block containing NADPH oxidase-3 (*Nox3*). Our peak was detected after an 8 kHz tone burst stimulus. *Nox3* mutants and heterozygotes were then tested to validate our GWAS. The mutants and heterozygotes demonstrated a greater susceptibility to NIHL specifically at 8 kHz both on measures of distortion product otoacoustic emissions (DPOAE) and on auditory brainstem response (ABR). We demonstrate that this sensitivity resides within the synaptic ribbons of the cochlea in the mutant animals specifically at 8 kHz. Our work is the first GWAS for NIHL in mice and elucidates the power of our approach to identify tonotopic genetic susceptibility to NIHL.

## Author Summary

Noise-induced hearing loss (NIHL) is the most common work-related disease in the world and the second cause of hearing loss. Although several candidate gene association studies for NIHL in humans have been conducted, each are underpowered, un-replicated, and account for only a fraction of the genetic risk. Buoyed by the prospects and successes of human association studies, several groups have proposed mouse genome-wide association studies. The environment can be carefully controlled, facilitating the study of complex traits like NIHL. In this manuscript, we describe, for the first time, an association analysis with correction for population structure for the mapping of several loci for susceptibility to NIHL in inbred strains of mice. We identify *Nox3* as the associated gene for susceptibility to NIHL that the genetic susceptibility is frequency specific and that it occurs at the level of the cochlear synaptic ribbon.

## Introduction

Noise-induced hearing loss (NIHL) is a worldwide leading occupational health risk in industrialized countries and is the second most common form of sensorineural hearing impairment, after presbyacusis [1]. In the United States, roughly 10% of the total population is exposed daily to hazardous levels of noise in the workplace [2]. The most extreme workplace environment for NIHL is the Armed Forces. According to the Department of Veterans Affairs, hearing loss is the most common disability among U.S. troops in the Middle East. The financial impact of these disability claims on the VA is staggering and likely will continue to grow. According to the American Tinnitus Association (http://www.ata.org/), the number of disability claims from hearing injury is expected to increase by 18% per year with a total cost of $1.2 billion annually [3]. Risk could be reduced with a better understanding of the biological processes that modulate susceptibility to damaging noise. It is believed that NIHL is a complex disease resulting from the interaction between environmental and genetic factors and it is well recognized that people with similar exposures to noise show variation in the amount of hearing loss, indicative of a genetic component [4].Twin studies estimate heritability for noise-induced hearing loss (NIHL) of approximately 36% [5].

The discovery of gene by environment interactions in human disease, such as susceptibility to NIHL, has many inherent difficulties, most notably, controlling for exposure. Although several candidate gene association studies for NIHL in humans have been conducted, each is underpowered, un-replicated, and accounts for only a fraction of the genetic risk. In addition, no heritability studies have been performed, since families, where all subjects are exposed to identical noise conditions, are almost impossible to collect.

The genetic basis of NIHL has been clearly demonstrated in animals as different susceptibilities to noise have been seen in different inbred stains of mice [4]. Mouse strains (C57BL/6J) exhibiting age-related hearing loss (AHL) were shown to be more susceptible to noise than other strains [6]. Also, several knockout mice including SOD1-/- [7], GPX1-/- [8], PMCA2-/- [9] and CDH23+/- [10] were shown to be more sensitive to noise than their wild-type littermates. The mouse has been an essential animal model for studies in hearing loss, and advances in mouse genetics, including genome sequence and high density single-nucleotide polymorphism (SNP) maps, provide a suitable system for the study of a complex trait such as NIHL [6]. The identification of novel genes is crucial for the discovery of new pathways and gene networks that will improve our knowledge of basic hearing biology and identify new therapeutic targets with the potential to combat NIHL.

Due to the limitations of human genome-wide association study (GWAS) and quantitative trait locus (QTL) analyses in mice, we have chosen to use a genome-wide association strategy incorporating the Hybrid Mouse Diversity Panel (HMDP). The HMDP is a collection of classical inbred (CI) and recombinant inbred (RI) strains whose genomes have been sequenced and/or genotyped at high resolution [11]. Power calculations have demonstrated that this panel is superior to traditional linkage analysis and is capable of detecting loci responsible for 5% of the overall variance. Several studies have successfully mapped candidate loci for complex traits using this panel and we have recently published a meta-analysis for age-related hearing loss incorporating the HMDP [12] [13] [14] [15].

In this manuscript we describe, for the first time, an association analysis with correction for population structure in the mapping of several loci for susceptibility to NIHL in inbred strains of mice. After completing a preliminary screen of the HMDP, an intriguing locus appeared warranting further exploration. Herein, we describe a genome-wide significant peak on (Chr.) 17 within a haplotype block containing NADPH oxidase-3 (*Nox3*) and provide evidence supporting its role in susceptibility to NIHL. Furthermore, we demonstrate frequency-specific genetic susceptibility within the mouse cochlea.

## Methods

### Ethics Statement

The Institutional Care and Use Committee (IACUC) at University of Southern California, Los Angeles, approved the animal protocol for the HMDP strains and the *Nox3* ^*het*^ mice (IACUC 12033). HMDP strains and C57BL/6JEiJ *Nox3* ^*het*^ (*Nox3* ^*het*^/*Nox3* ^*het*^, *Nox3* ^*het*^/+ and wild-type) were anesthetized with an intraperitoneal injection of a mixture of ketamine (80 mg/kg body weight) and xylazine (16 mg/kg body weight).

### The Hybrid Mouse Diversity Panel

A detailed description of the HMDP (strain selection, statistical power and mapping resolution) is provided in Bennett BJ, et al. 2010. [11]. Approximately four female mice for each HMDP strain were purchased from the Jackson Laboratory (Bar Harbor, ME). Only female mice were tested to avoid confounding effects of sex. Mice were 4 weeks of age, and to ensure adequate acclimatization to a common environment, mice were aged until 5 weeks. 5-week-old mice were selected to eliminate the potential effects of age-related hearing loss contributing to our phenotype. All mice were maintained on a chow diet until sacrifice.

### Genotyping

Common and recombinant inbred strains were previously genotyped by the Broad Institute (www.mousehapmap.org). Of the 140,000 SNPs available, 108,064 were informative (allele frequency ≥ 5% and less than 20% missing data) and were used for the association analysis.

### Pre and Post Noise Exposure Hearing Thresholds

Stainless-steel electrodes were placed subcutaneously at the vertex of the head and the right mastoid, with a ground electrode at the base of the tail. Body temperature was maintained and monitored. Artificial tear ointment was applied to the eyes. Each mouse was recovered on a heating pad at body temperature. Auditory signals were presented as tone pips with a rise and a fall time of 0.5 msec and a total duration of 5 msec at the frequencies 4, 8, 12, 16, 24, and 32 kHz. Tone pips were delivered below threshold and then increased in 5 dB increments until goal of 100 dB. Signals were presented at a rate of 30/second. Responses were filtered with a 0.3 to 3 kHz pass-band (x10,000 times). For each stimulus intensity 512 waveforms were averaged. Hearing threshold was determined by inspection of auditory brainstem response (ABR) waveforms and was defined as the minimum intensity at which wave 1 could be distinguished. Data was stored for offline analysis of peak-to-peak (P1-N1) values for wave 1 amplitudes. Post-exposure thresholds were evaluated by the same method 2 weeks post-exposure.

### Pre and Post Exposure DPOAE Determination

Distortion product otoacoustic emissions (DPOAEs) were analyzed as input/output (I-O) functions with 2f_1_- f_2_ (primary measure). Primary tones were set at a ratio of f_2_/f_1_ = 1.2 with the f_2_ between 8 to 32 kHz(f_2_ level set 10 dB less than the f_1_ level)and L_2_ ranging from 20 to 70 dB. The noise floor was measured by averaging 6 spectral points (above and below the 2f_1_- f_2_). After both waveform and spectral averaging DPOAEs were extracted. Threshold was defined as the L_2_ level needed to produce a DPOAE of 0 dB SPL with a signal to noise ratio (SNR) ≥ 3 dB.

### Noise Exposure and Audiometric Equipment

6 week-old mice were exposed for 2 hours to 10 kHz octave band noise (OBN) at 108 dB SPL using a method adapted from Kujawa and Liberman (2009) [16]. The OBN noise exposure was previously described [17]. For 2 hours, mice were placed in a circular ¼-inch wire-mesh exposure cage with four shaped compartments and were able to move about within the compartment. The cage was placed in a MAC-1 soundproof chamber designed by Industrial Acoustics (IAC, Bronx, NY) and the sound chamber was lined with soundproofing acoustical foam to minimize reflections. Noise recordings were played with a Fostex FT17H Tweeter Speaker built into the top of the sound chamber. Calibration of the damaging noise was done with a B&K sound level meter with a variation of 1.5 dB across the cage.

A data acquisition board from National Instruments (National Instruments Corporation, Austin, Texas) was regulated by custom software (used to generate the stimuli and to process the responses). Stimuli were provided by a custom acoustic system, made up of two miniature speakers, and sound pressure was measured by a condenser microphone. Testing involved the right ear only. All hearing tests were performed in a separate MAC-1 soundproof chamber to eliminate both environmental and electrical noise.

### Cochlear RNA Extraction

For each HMDP strain, both cochleae from each 8-week-old mouse were removed. The inner ear was micro-dissected and the surrounding soft tissue and the vestibular labyrinth was removed. The dissected cochleae were then frozen in liquid nitrogen and then ground to powder. RNA was extracted and purified by placing cochlea samples in RNA lysis buffer (Ambion). The sample was incubated overnight (4^°^C), centrifuged (12,000g for 5 minutes) to pellet insoluble materials and RNA isolated (following manufacturer’s recommendations). This procedure generates approximately 300 ng of total RNA per mouse.

### Gene Expression Analysis

Illumina’s Mouse whole genome expression, BeadChips, was used for the gene expression measurements. Amplifications and hybridizations were performed according to Illumina’s protocol (Southern California Genome Consortium microarray core laboratory at UCLA). RNA was reverse transcribed to cDNA using Ambion cDNA synthesis kit (AMIL1791) and then converted to cRNA and labeled with biotin. Further, 800ng of biotinylated cRNA product was hybridized to prepare whole genome arrays and was incubated overnight (16–20 hrs) at 55^°^C. Arrays were washed and then stained with Cy3 label. Excess stain was removed by washing and then arrays were scanned on an Illumina BeadScan confocal laser scanner.

### Efficient Mixed-Model Association (EMMA)

EMMA is a statistical test for association mapping correcting for genetic relatedness and population structure and consider the mean per strain and also individual measurement per mouse to increase the statistical power. We have previously demonstrated that p <0.05 genome-wide equivalent for GWA using EMMA in the HMDP is P = 4.1×10^-6^ (−log10P = 5.39)[18]. An R package implementation of EMMA is available online at http://mouse.cs.ucla.edu/emma.

### Candidate Gene Characterization

RefSeq genes were downloaded from the UCSC genome browser (https://genome.ucsc.edu/cgi-bin/hgGateway) using the NCBI Build37 genome assembly to characterize genes located in each association. EMMA was used to calculate association (P-values) for the probes corresponding to the RefSeq genes. The confidence interval (95%) for the distribution of distances between the most significant and the true causal SNPs, for simulated associations that explain 5% of the variance in the HMDP, is 2.6 Mb [11]. Only SNPs mapping to each associated region were used in this analysis. We selected SNPs that were variant in at least one of the HMDP classical inbred strains. Non-synonymous SNPs within each region were downloaded from the Mouse Phenome Database (http://phenome.jax.org/).

### Characterizing the *Nox3* ^*het*^ Mice

The generation and initial characterization of *Nox3* ^*het*^ allele was previously described [19]. The *Nox3* ^*het*^ allele arose spontaneously (endogenous retroviral insertion into intron 12) on the GL/Le strain, but has since been made congenic onto the C57BL/6JEiJ strain. To circumvent the probability of additional alleles from the donor strain this congenic region was backcrossed for more than 10 generations. Since the *downless* mutant allele is not present in this strain the congenic interval containing *Nox3* is likely less than 5 centimorgans (http://jaxmice.jax.org/strain/002557.html). *Nox3* ^*het*^ (known as the *head-tilt* or *het* mice) carry autosomal recessive, spontaneous mutations that lead to otoconial absence with no apparent abnormalities in other organs. The otoconia deficit results in head-tilting behavior and absent vestibular-evoked potentials (VsEPs) but normal thresholds ABR [20].

Pre exposure ABR, DPOAE and VsEP in male and female mice (5 weeks old) of varying *Nox3* ^*het*^ genotype (*Nox3* ^*het*^/*Nox3* ^*het*^ and *Nox3* ^*het*^/+) and wild-type (C57BL/6JEiJ strain) was measured as described above. Pre-exposure threshold levels were obtained at 1 week prior to noise exposure and the animals were assessed for noise damage 2 weeks after exposure. The ABR permanent threshold shift (PTS) was defined as the difference between pre-exposure and post-exposure thresholds at each tested frequency. One-way ANOVA was used to test the significance and *post hoc* Tukey test for multiple comparisons.

### Cochlear Whole Mount Preparation

Mice were sacrificed less than 24 hours after the post exposure ABR. Cochleae were dissected from the surrounding tissues and openings were made into the coils by piercing the apex and rupturing both the oval and the round windows. The dissection was done in cold PBS. After dissection, cochleae were fixed in 4% paraformaldehyde for overnight at 4^°^C and then washed with PBS. Further dissection was done to expose the organ of Corti. For permeabilization and blocking, tissue was immersed for 1 hour in PBS containing 0.2% Triton X-100 (Sigma Chemical) and 16% normal goat serum (SouthernBiotech). Samples were incubated overnight at room temperature with primary antibodies (rabbit anti-myosin6, 1:500, Proteus Biosciences and purified mouse anti-CtBP2, 1:500, BD Biosciences) for doubled-staining. Secondary antibody was then applied and tissue was incubated in the dark overnight (Alexa 594 donkey anti-rabbit, 1:500, Life technologies and Alexa Fluor-488 anti-mouse, 1:500, Life technologies). After, samples were washed three times in PBS and mounted on glass slides using Fluoromount G (SouthernBiotech). Microscopy was carried out with a laser confocal microscope (Olympus IX81) with epifluorescence light (Olympus Fluoview FV1000). Outer hair cell loss (% per 100μm) was counted and plotted as cytocochleogram by relating distance of cochlear apex to the tonotopic map of mice of strain CBA [21]. Percentages indicate the normalized location of the inner and outer hair cells in the cochlea (0%, apical and 100%, basal end) in 10% steps.

Synaptic ribbon density was plotted for each correspondent ABR frequency (4, 8, 12, 16, 24 and 32 kHz) against the same tonotopic map. Inner hair cells were analyzed in a row (50 μm) for each frequency. CtBP2 immunofluorescence spots were counted in z-stacks and divided by the number of inner hair cells (measured as the quantity of nuclei) in the sample.

### *Nox3* ^*het*^ PCR

Polymerase chain reaction (PCR) was performed for *Nox3* using the following primers: *Nox3*-int12F, GTTCTGGAGCACCACCTTGT; *Nox3*-int12R CCCATAGGGAGCCAAGAAAT; and ERV-R, TGTCAAGCTGACTCCACCAG [19]. PCR products were separated on a 1.5% agarose gel containing 0.5 mg/ml ethidium bromide.

## Results

### There Exists Phenotypic Variation in Susceptibility to NIHL within the HMDP

In an effort to identify genomic regions associated with NIHL susceptibility, we phenotyped 5-week old female mice (n = 297) from 64 HMDP strains (n = 4–5/strain) for thresholds after noise exposure using Auditory Brainstem Response thresholds at specific ABR stimulus frequencies. The stimuli consisted of 4, 8, 12, 16, 24 and 32 kHz tone bursts.

**Fig 1:**
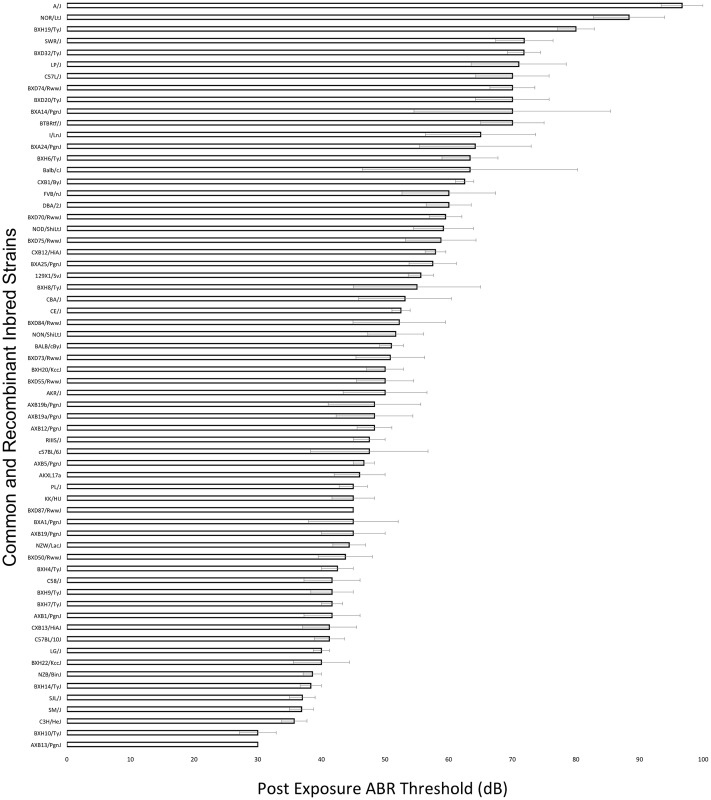
Characterization of post-exposure thresholds in the HMDP. Mean ± SEM for 8 kHz post-noise exposure hearing thresholds in 64 HMDP inbred mouse strains. The difference between the strains with the lowest and the highest values were 3.22-fold.

A wide range of ABR thresholds were observed across the HMDP with differences of 3.22-fold between the lowest and the highest strains for thresholds at 8 kHz post-noise exposure (Fig. 1). Frequencies of 4, 12, 16, 24 and 32 kHz demonstrated differences of 1.55, 3.25, 3.57, 2.74 and 3.75-fold, respectively.

### Genome-Wide Association Analysis of NIHL Reveals Frequency Specific Genetic Susceptibility

EMMA algorithm was applied to each phenotype separately to identify genetic associations for the six tone-burst stimuli [18]. Adjusted association p-values were calculated for 108,064 SNPs with minor allele frequency of > 5% (p < 0.05 genome-wide equivalent for GWA using EMMA in the HMDP is p = 4.1 x 10^-6^,-log10P = 5.39).

**Fig 2:**
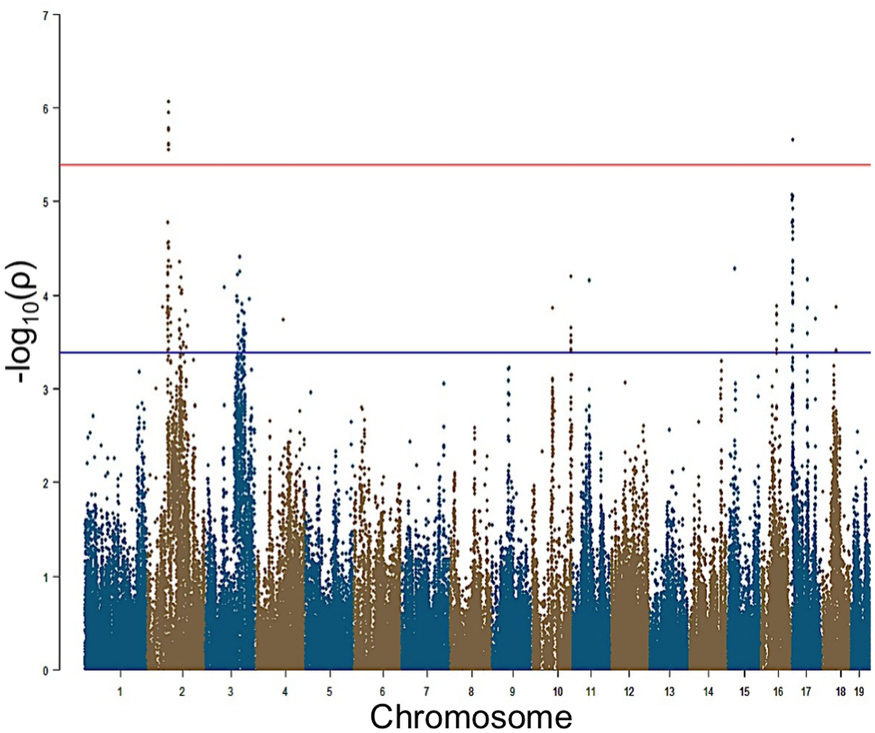
GWAS results for post-noise exposure thresholds in the HMDP. Manhattan plot showing the association (-log10) p-values (-logP) for 8 kHz in 64 HMDP inbred mouse strains. The analysis was performed using 108,064 SNPs with a minor allele frequency > 5%. Each chromosome is plotted on the x-axis in alternating brown and blue colors. SNPs on Chr. 2 and Chr. 17 for 8 kHz exceeded the predetermined genome-wide significance threshold (-logP = 5.39).

At this threshold, genome-wide significant associations on Chr. 2 (rs27972902; p = 8.6x10^-7^) and Chr. 17 (rs33652818; p = 2.3x10^-6^) were identified for the 8 kHz stimuli (Table 1, Fig. 2). Additionally, a significant association signal on Chr. 15 (rs32934144; p = 1.7x10^-6^) was identified for the 16 kHz tone burst and two significant regions on Chr. 3 (rs30795209; p = 5.5x10^-7^) and Chr. 15 (rs32278602; p = 5.9x10^-7^) were identified at 32 kHz.

**Table 1:** GWA results for NIHL in the HMDP.

Trait^a^	Chr	SNP	Position (Mb)^b^	-logP	MAF^c^	No. Of Genes^d^	Human Region (Chr: Start Mb—End Mb)
**4 kHz**	17	rs33652818	3.8	1.1E-04	0.222	10	Chr6:155.0–155.7
**8 kHz**	2	rs27972902	68.1	8.58E-07*	0.222	2	Chr2:168.8–169.6
**8 kHz**	17	rs33652818	3.8	2.25E-06*	0.222	10	Chr6:155.0–155.7
**16 kHz**	15	rs32934144	28.2	1.74E-06*	0.389	2	Chr5:13.6–14.8
**32 kHz**	3	rs30795209	96.7	1.95E-07*	0.278	11	Chr1:145.6–147.4
**32 kHz**	15	rs32278602	22.3	5.86E-07*	0.389	2	Chr5:19.4–22.2

^a^Post noise-exposure hearing thresholds in different frequencies.

^b^Locations based on genome assembly (NCBI´s Build37)

^c^MAF, minor allele frequency

^d^Number of RefSeq genes (NCBI´s Build37 assembly) located in the mouse association confidence interval (2.6 Mb)

* Genome-wide significant (p<4.1E-6)

### Characterization of NIHL GWAS Peaks

Within each association peak there were 4 (Chr. 15), 11 (Chr. 3), 10 (Chr. 17) and 2 (Chr. 2) unique RefSeq genes. We next identified genes within each of the five intervals possessing functional alterations. Genes were selected based upon their regulation by a local expression QTL (eQTL) in the HMDP or if they harbored a non-synonymous (NS) SNP that was predicted to have functional consequences. For the eQTL analysis, we generated gene expression microarray profiles using RNA isolated from cochleae in 64 HMDP strains (n = 3 arrays per strain). EMMA was then used to perform an association analysis between all SNPs and array probes mapping within each region. A total of 18,138 genes were represented by at least one probe, after excluding probes that overlapped SNPs, present among the classical inbred strains used in the HMDP (see Methods). Of these, 6 genes (4 within Chr. 3 association and 2 within Chr. 17 association) were identified with at least one probe whose expression was regulated by a local eQTL (Table 2). However, the only probe whose expression was regulated by a significant local eQTL in the cochlea was located on Chr. 17.

**Table 2:** Genes within NIHL 5 association peaks regulated by local eQTL in the cochlea.

Gene	RefSeq	Chr	txStart (bp)^a^	txEnd (bp)^b^	Local eQTL P^c^
**Pias3**	ILMN_2631014	3	96696384	96706070	6.78E-02
**CD160**	ILMN_2707181	3	96798763	96829351	1.49E-02
**Gja8**	ILMN_2625168	3	96918863	96926020	3.09E-01
**Gja5**	ILMN_2678477	3	97032416	97053634	4.07E-01
**Tiam2**	ILMN_2836875	17	3326573	3519397	3.71E-01
**Tfb1m**	ILMN_2690441	17	3519263	3557713	1.08E-06

^a^txStart, location of transcription (NCBI Build37 genome assembly) start.

^b^txEnd, location of transcription (NCBI Build37 genome assembly) end.

^c^Statistically significant p value ≤ 5.1E-04 (Bonferroni corrected for the number of probes tested)

We determined whether any of the 27 genes implicated in our preliminary GWAS had a defined role in the inner ear. The associations on Chr. 2, 3 and 15 did not harbor known cochlear genes. Only NADPH oxidase 3 (*Nox3*) on Chr. 17 had been implicated in inner ear biology with mutants lacking otoconia in the utricular and saccular maculae [22] and its high expression in the inner ear [23].

### Detailed Analysis of the Chr 17 Association Highlights *Nox3* as a Candidate Gene

**Fig 3:**
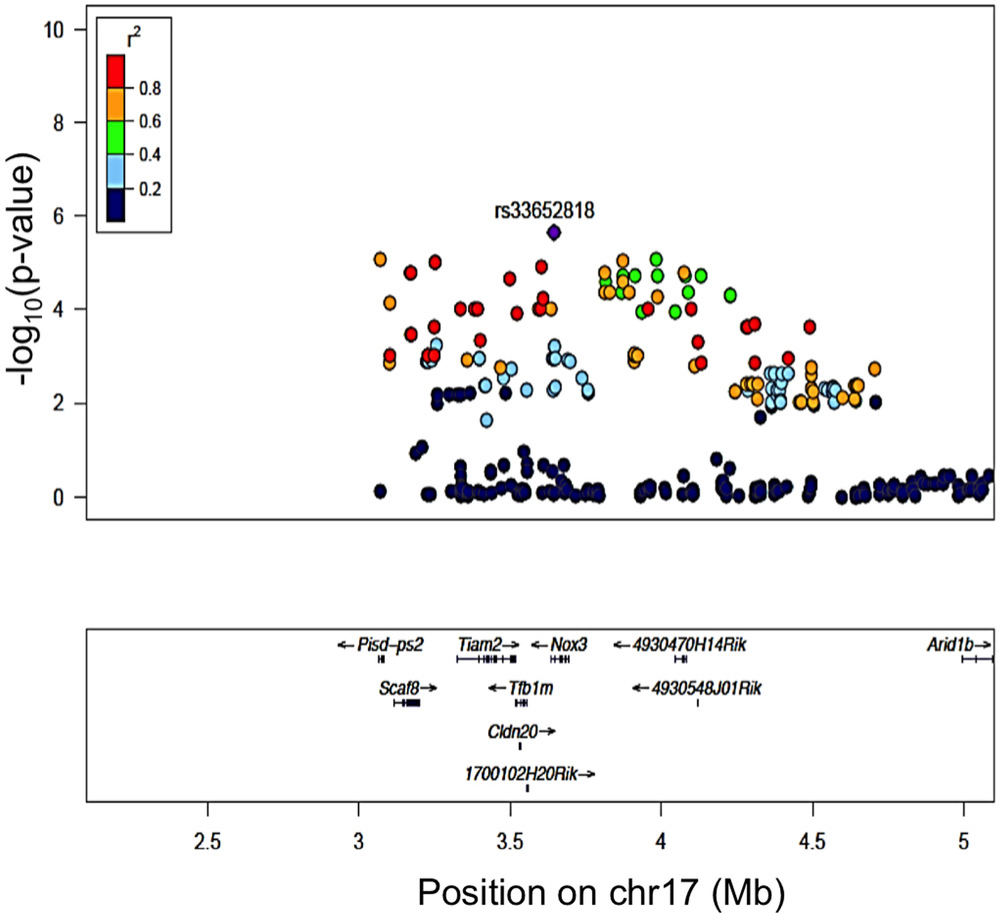
Regional plot of the 8 kHz ABR post noise-exposure at Chr 17 association in the HMDP centered on the lead SNP at the Nox3 locus (rs33652818). The blue diamond represents the most significant SNP (p = 9.63E-06) and SNPs are colored based on their LD with the most significant SNP being: red SNPs in LD at r^2^>0.8, orange SNPs in LD at r^2^>0.6 and green SNPs in LD at r^2^>0.4. The positions of all RefSeq genes are plotted using genome locations (NCBI’s Build37 genome assembly).

Of all genes at the chromosome 17 locus, one gene, *Tfb1m*, had a significant (1.08x10^-6^) eQTL (Fig. 3). Of note, *Nox3*, the gene in which our peak GWAS SNP is located, does not have an eQTL in the cochlea; however, there was a clear demonstration [23] that *Nox3* is highly expressed (at least 50-fold higher than in any other tissues) in specific portions of the inner ear. Based on these data and the location of our peak GWAS SNP (rs33652818), we focused on *Nox3* as a plausible candidate gene for NIHL at the chromosome 17 locus.

### *Nox3* ^*het*^ Mice Are More Susceptible to NIHL

**Fig 4:**
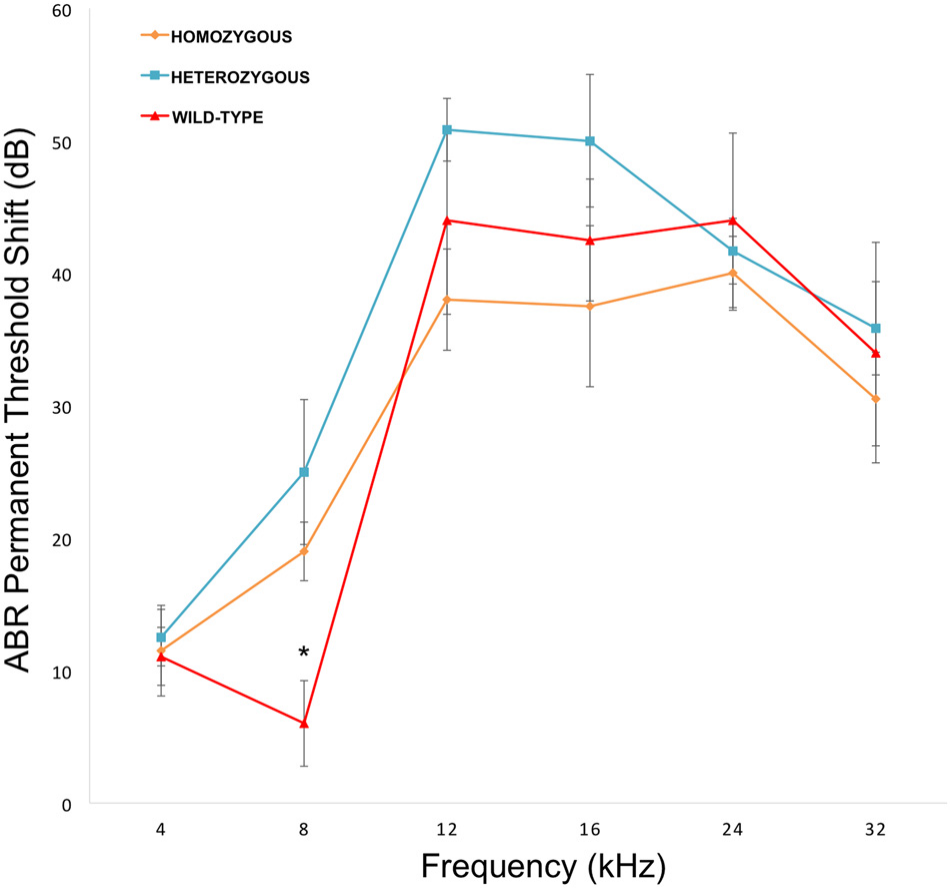
*Nox3* ^het^ mice have greater PTS (permanent threshold shift) for 8 kHz. *Nox3* ^*het*^/*Nox3* ^*het*^ and *Nox3* ^*het*^/+ display significantly greater PTS in comparison to wild-type controls. Data shown are mean comparisons analyzed by one-way ANOVA (post hoc Tukey test for multiple comparisons). *p < 0.05. Homozygous = *Nox3* ^*het*^/*Nox3* ^*het*^: Heterozygous = *Nox3* ^*het*^/+: Wild-type = C57BL/6JEiJ strain.

**Fig 5:**
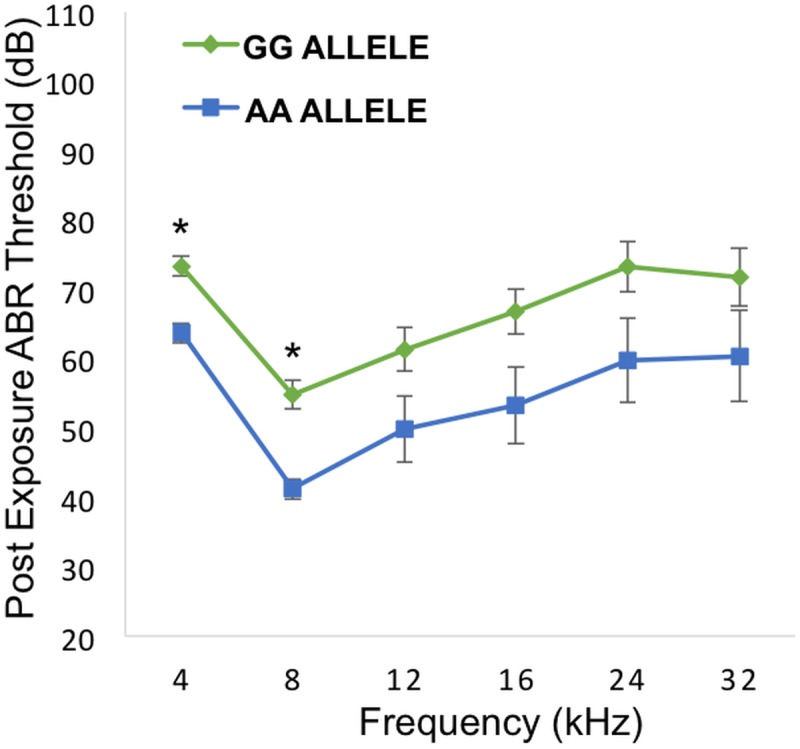
Genotypic effects of the peak SNP (rs33652818) at the *Nox3* locus. Comparison between alleles GG and AA across the various frequencies. There is a statistically significant difference between the alleles at 4 and 8 kHz. * = p value < 0.001. Error bars +/- 1 SE.

To directly test the hypothesis that *Nox3* was associated with susceptibility to NIHL we characterized previously generated *Nox3* ^*het*^ mice for pre- and post-noise exposure ABR thresholds and PTS after 4, 8, 12, 16, 24 and 32 kHz tone-burst stimuli. Consistent with our original GWAS finding, this analysis revealed a statistically significant reduction in the PTS in wild-type mice (C57BL/6JEiJ strain) compared to *Nox3* ^*het*^/^*+*^ and *Nox3* ^*het*^/*Nox3* ^*het*^ at 8 kHz (Fig. 4). As a comparison, the effects of the peak SNP (rs33652818) at the *Nox3* locus on ABR at various frequencies is shown in Fig. 5. Interestingly, there were significant differences as a function of genotype at both the 4 kHz and the 8kHz test frequencies, although the level of significance at 4 kHz (p = 1.1x10^-4^) is only suggestive (S1 Fig) and does not reach genome-wide significance (Table 1). Thus, the significant and highly suggestive association of rs33652818 with ABR at 8 and 4 kHz, respectively, in the HMDP, as well as the frequency-specific phenotype exhibited by the *Nox3* ^*het*^/*Nox3* ^*het*^ mice, suggests that *Nox3* may be involved in NIHL at the lower end of the frequency spectrum.

**Fig 6:**
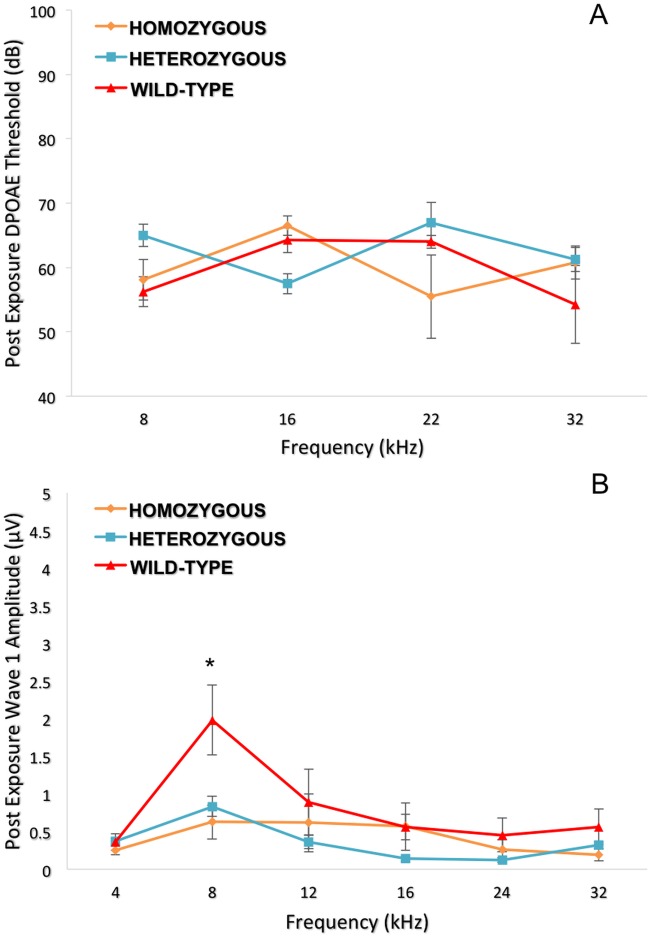
Topographical analysis of the auditory pathway at different frequencies (post-noise exposure). Although no significant difference was seen for the DPOAE thresholds (6A), the wild-type controls show a higher wave 1 amplitude (p = 0.010) only at 8 kHz compared to *Nox3* ^*het*^/+ and *Nox3* ^*het*^/*Nox3* ^*het*^ (6B). One-way ANOVA (Tukey test for multiple comparisons). * = p < 0.05. Homozygous = *Nox3* ^*het*^/*Nox3* ^*het*^: Heterozygous = *Nox3* ^*het*^/+: Wild-type = C57BL/6JEiJ strain.

For a detailed analysis of the entire auditory pathway, we next evaluated outer hair cell (OHC) activity using DPOAE and the inner hair cell (IHC) and neuronal responses by ABR wave I peak-to-peak amplitudes. Despite the absence of a statistically significant difference in DPOAE thresholds (Fig. 6A) at 8, 16, 22 and 32 kHz, there was a pronounced difference at 8 kHz in the wave 1 ABR peak-to-peak amplitudes (Fig. 6B).

**Fig 7:**
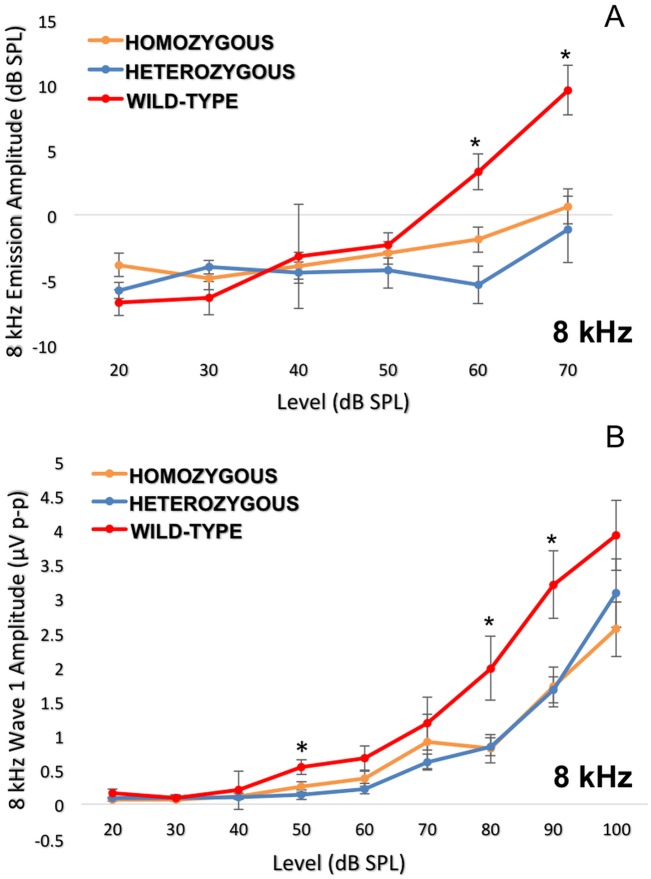
Detailed analysis of the 8 kHz frequency stimulus. Dissection of the 8 kHz frequency by DPOAE I/O function (7A) and ABR wave 1 amplitudes (7B) consistently indicate more impairment in the *Nox3* ^*het*^/*Nox3* ^*het*^ and *Nox3* ^*het*^/+ than wild-type. One-way ANOVA (Tukey multiple comparisons). * = p < 0.05. Homozygous = *Nox3* ^*het*^/*Nox3* ^*het*^: Heterozygous = *Nox3* ^*het*^/+: Wild-type = C57BL/6JEiJ strain.

The DPOAE (Fig. 7A) suprathreshold amplitudes (dB SPL) and ABR wave 1 amplitudes (μV) (Fig. 7B) for the 8 kHz tone burst were compared at different stimulus intensities. Both analyses demonstrated statistically significantly less noise damage in the wild-type in comparison to the heterozygous and mutant mice.

**Fig 8:**
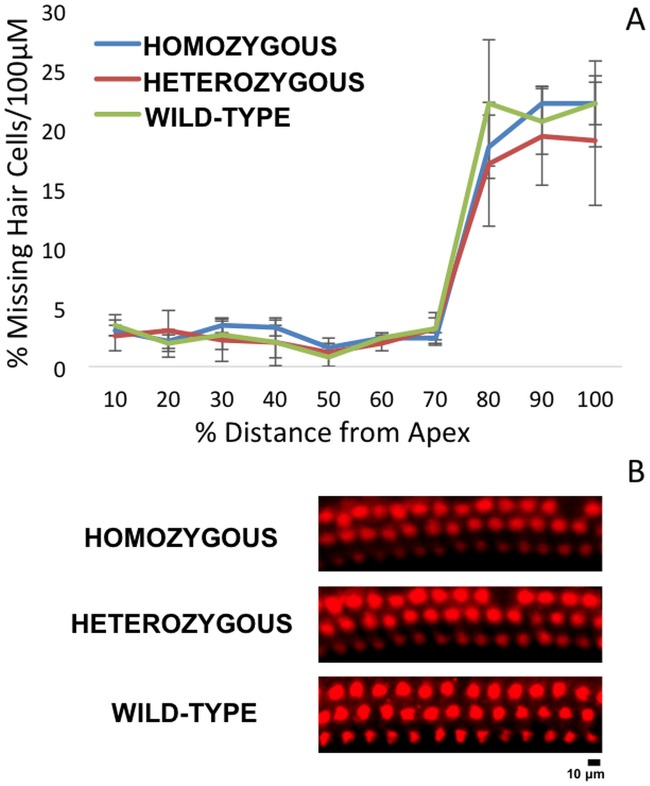
Cytocochleogram of wild-type and mutant *Nox3* mice (8A). No significant difference in OHC counts were detected among wild-type, *Nox3* ^*het*^/+ and *Nox3* ^*het*^/*Nox3* ^*het*^. OHC preparations of immunostained (40x) post-noise exposure cochleae (8B) at the 8 kHz tonotopic location (red, rabbit anti-myosin6) demonstrate a small impact with the loss of apical OHC (2 weeks post-noise exposure). Homozygous = *Nox3* ^*het*^/*Nox3* ^*het*^: Heterozygous = *Nox3* ^*het*^/+: Wild-type = C57BL/6JEiJ strain.

**Fig 9:**
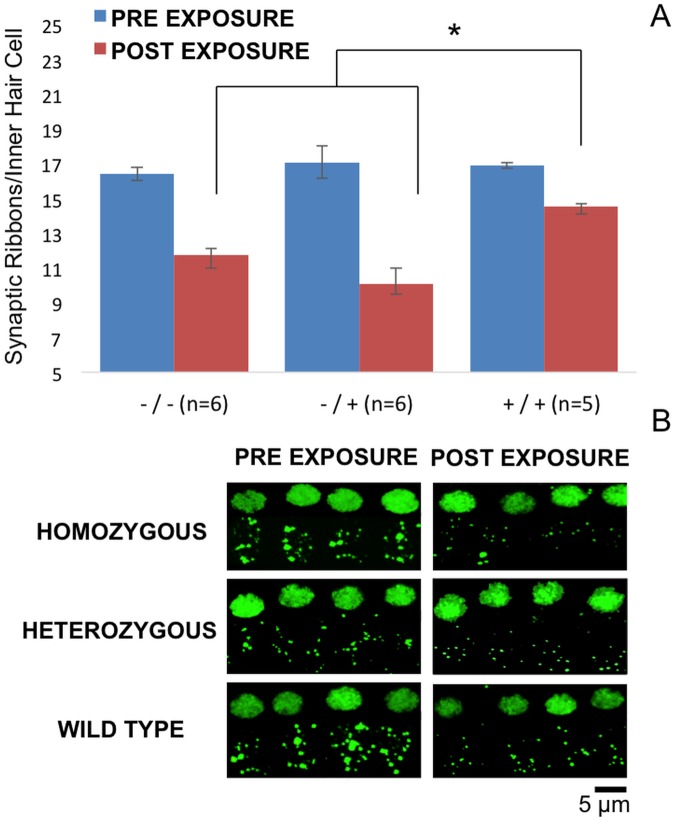
8 kHz synaptic cochleogram. Synaptic ribbon count measured at the 8 kHz tonotopic position within the cochlea. Despite the absence of a statistical difference in OHC counts, the wild-type mice demonstrated significantly greater post-noise exposure synaptic ribbon density per IHC (9A). Projections (60x, 3x zoom, oil immersed) of confocal stacks (9B) of immunostained pre and post-noise exposure mouse IHC synaptic ribbons (green, mouse anti-CtBP2).-/- = *Nox3* ^*het*^/*Nox3* ^*het*^:-/+ = *Nox3* ^*het*^/+: +/+ = wild-type.

To confirm these electrophysiological findings, we collected cochleae from pre- and post-noise exposure *Nox3* ^*het*^ mice and wild-type. First, we assessed OHC loss throughout the entire cochlea by creating a cytocochleogram (Fig. 8A) of immunolabeled (Fig. 8B) whole-mount organs of Corti to correlate with the DPOAE findings. Subsequently, the IHC afferent synaptic density (Fig. 9) was analyzed as a marker of the neuronal responses (suprathreshold ABR wave 1 amplitude).

Despite the absence of a statistical significance in OHC loss, the *Nox3* ^*het*^/+ and *Nox3* ^*het*^/*Nox3* ^*het*^ mice demonstrated a significantly reduced post-noise exposure density of synaptic ribbons (at the 8kHz tonotopic location).

## Discussion

### NIHL Genome-Wide Association Study

We have, for the first time, used association analysis with correction for population structure to map several loci for hearing traits in inbred strains of mice. Our results identify a number of novel loci for susceptibility to NIHL. Additionally, our study demonstrates frequency-specific genetic susceptibilities to noise within the cochlea and the power of our GWAS to detect frequency-specific loci that are precisely recapitulated in a mutant mouse model.

Mouse GWAS has revolutionized the field of genetics and has lead to the discovery of hundreds of genes that are involved in complex traits [24]. Our successful mapping largely came from the initial observation that there was a clear strain variation at all post noise exposure hearing phenotypes, reiterating the contribution of genetic factors to NIHL susceptibility. This wide distribution of phenotypes and genotypes facilitated our high-resolution genetic mapping.

We used a combined set of 64 classic inbred and recombinant inbred strains, a portion of the HMDP, as an extension of the classical inbred strain association. This increased the statistical power of the classical association studies by including a set of recombinant inbred strains in the mapping panel [25]. The HMDP provided significant statistical power and resolution to identify a locus for NIHL susceptibility that was precisely modeled in a mutant strain [26]. Although this panel is composed of 100 commercially available inbred strains, with roughly two-thirds of this panel we were able to map 5 loci, reflecting the power to detect loci with moderate effect. In addition to the power present in this resource, the resolution of this panel is, in some cases, two orders of magnitude better than that achieved with linkage analysis, as we have recently demonstrated in our mouse GWAS for age-related hearing loss [27].

In an unprecedented manner, this new paradigm was applied to the first high-resolution mapping of candidate genes for NIHL susceptibility. Our GWAS generated significant associations in at least five loci at three different post-noise exposure stimulus frequencies, corresponding to a total 27 candidate genes. All of these candidate genes require adequate characterization, but the first gene to be validated by a genetic mutant mouse model was *Nox3*. *Nox3* was selected for further investigation based upon its relatively restricted expression in the cochleo-vestibular epithelium and spiral ganglion neurons [23].

### *Nox3* and the Inner Ear

The *Nox3* gene was described in 2000 based upon its sequence similarity to other *Nox* isoforms (encodes an NADPH oxidase) [28]. The overall structure of *Nox3* is highly similar to that of *Nox1* and *Nox2* [29] and *Nox3* shares 56% amino acid with *Nox2* [30]. Encoded by *Nox3*, the six-transmembrane NADPH-binding protein interacts with a two-transmembrane protein (encoded by *Cyba*) and a cytosolic protein (encoded by *Noxo1*). This activation releases a functional NADPH oxidase complex that is able to transporting electrons across membranes towards oxygen (O_2_) generating superoxide (O_2_•-) and subsequent reactive oxygen species (ROS) [19].

First studies on the *Nox3* function were published in 2004 and generated the definition of *Nox3* as an NADPH oxidase of the inner ear [23][22]. Banfi, et al., performed analysis of *Nox3* distribution (real time PCR and *in situ* hybridization) and reported high *Nox3* expression in the inner ear (cochlear/vestibular sensory epithelia and the spiral ganglion). Following exposure to cisplatin, HEK293 cells transfected with *Nox3* produced O_2_•- spontaneously and generated a dramatic increase in O_2_•- production [23]. Paffenholz et al. [22] reported that mutations of the *het* locus affect *Nox3* and that these head tilt mice (*het*) have impaired otoconial formation in the utricle and saccule resulting in balance defects, such as the inability to detect linear acceleration or gravity. Based upon this finding we chose to pursue interrogation of *Nox3*, a gene within our locus on Chr. 17.

Subsequent studies have established a role for the *Nox3* gene as the primary source of ROS generation in the cochlea, especially induced by cisplatin ototoxicity [31]. The knockdown of *Nox3* (pretreatment with siRNA) prevented cisplatin ototoxicity with preservation of hearing thresholds and hair cells. Also, it reduced the expression of *Nox3* and biomarkers of damage (TRPV1 and KIM-1) in cochlear tissues [32]. siRNA-mediated gene silencing of *Nox3* alleviated cisplatin-induced hearing loss in rats and reduced apoptosis of the sensory hair cells in the cochlea [33]. Although there was no similar evidence regarding NIHL, this key role for *Nox3* in the development of cisplatin ototoxicity confirming its role in regulatory mechanisms of cochlear damage encouraged us to validate this candidate gene for NIHL.

The only study exploring NIHL and the NOX family (including *Nox3*) was completed in rats [34]. This study did not indicate whether the *Nox3* gene decreased or increased the susceptibility to noise, but instead it evaluated *Nox3* expression levels after noise exposure. Some members of the NADPH oxidase family (*Nox1* and *Duox2*) were up-regulated in the rat cochlea after noise exposure, suggesting that these isoforms could be linked to cochlear injury. In contrast, the *Nox3* isoform was down-regulated after exposure to 100 dB SPL and 110 dB SPL by seven and fivefold respectively, which could represent an endogenous protective mechanism against oxidative stress. This protective mechanism may have decreased the impact of the noise among wild-type rats by reducing the expression of *Nox3* and decreasing the difference related to mutants. However, the *in vivo* data was based on the use of a non-specific Nox inhibitor that targeted multiple members of this enzyme without conclusively demonstrating that *Nox3* plays a role in NIHL. Our study, by contrast, has used animal models with naturally occurring genetic variation and specific genetic perturbation of *Nox3* to directly implicate this oxidative stress enzyme in hearing.

According to our study, noise exposure might have an opposite effect to cisplatin on *Nox3* expression, suggesting differential involvement of *Nox3* on noise and cisplatin-induced cochlear damage. Based upon this literature we hypothesized that the absence or reduction of the *Nox3* gene product, responsible for the production of ROS in the cochlea, would reduce susceptibility to noise and were startled by our findings. A review of the literature shows there are several key protective mechanisms attributed to the Nox family of genes. These mechanisms include: host defense and inflammation (ROS-dependent killing, inactivation of microbial virulence factors, regulation of pH and ion concentration in the phagosome and anti-inflammatory activity), regulation of gene expression (TNF-alpha, TGF-beta1 and angiotensin II), cellular redox potential, cellular signaling (inhibition of phosphatases, activation of kinases, regulation of ion channel*s* and Ca2+ signaling), oxygen sensing (kidney, carotid body and lungs), biosynthesis, regulation of blood pressure, cell growth, angiogenesis, differentiation and senescence [30]. These protective mechanisms may very well play a role in the findings of susceptibility to NIHL in the wild-type animals.

### Validation of *Nox3’s* Role in NIHL

We were able to validate our frequency-specific GWAS findings in isolation by studying *Nox3* ^*het*^ mutant mice. After noise exposure there was a statistically significant difference between the wild-type mice in comparison to the homozygous mutants and the heterozygotes on several measures of auditory function specifically and solely after the 8 kHz exposure. Contrary to the initial expectations, the presence of the *Nox3* gene was clearly protective against noise damage. Also we were able to demonstrate the genotypic effect of the peak SNP at the same GWAS phenotype at 8 kHz. We also show genotypic effect on 4 kHz, but this finding was only suggestive in GWAS and not confirmed in *Nox3* ^*het*^ mutants.

We dissected this phenotype in detail physiologically by assessing OHC function using DPOAEs and IHC/auditory nerve function using ABR. Although there was no statistically significant difference in DPOAE thresholds amongst the genotypes, there was a marked difference in the amplitude of wave 1 of the ABR after suprathreshold stimulation with the 8 kHz tone burst. This suggested that the mechanism of hearing loss, in relation to *Nox3*, resided in the spiral ganglion neurons and likely at 8 kHz along the cochlear place map.

There are many genes differentially expressed along the tonotopic axis of the cochlea, and this has been shown for *Nox3* [35]. It is likely that our frequency specific finding of variation in susceptibility to NIHL is the result of this tonotopic expression pattern.

Considering that all of the results pointed to the area of 8 kHz, we initiated a thorough electrophysiological and histological dissection at this particular frequency. The evaluation of the DPOAEs and suprathreshold wave 1 ABR amplitudes was performed at multiple stimulus intensity levels. For each study, the wild-type were more resistant to NIHL at only at 8 kHz. We performed immunohistochemistry two weeks after the noise exposure. Although the difference in OHC loss was not significant, we demonstrated a significantly higher density of synaptic ribbons in wild-type mice. Thus, the electrophysiological findings were verified by the immunohistochemistry, demonstrating that the presence of *Nox3* is protective at the neuronal level and that the sensory neural hearing loss after noise exposure occurred at this level of the peripheral auditory system.

The absence of differences in outer hair cell count was also verified by its corresponding electrophysiological measure of DPOAE thresholds. However, through the evaluation of DPOAE suprathreshold amplitudes, we were able to observe a statistically significant higher amplitude in the wild-type mice. These three different measures of the integrity of the outer hair cells (outer hair cell count, DPOAE thresholds and DPOAE suprathresholds amplitudes) have different sensitivity profiles to demonstrate the impact of noise. Probably DPOAE suprathreshold amplitude is the most sensitive measurement, since there is greater signal-noise ratio. This metric indicates that there is significantly less impact on the activity of the outer hair cells in wild-type mice.

### *Nox3* Plays a Protective Role in the Cochlea of Mice

Although *Nox3* is associated with production of O_2_•- in the inner ear, the *Nox* family has several physiological and potentially protective mechanisms. Definitely, this protective role explains the fact that the absence of *Nox3* increased susceptibility to NIHL in our mouse models. However, there is a lack of specific studies about the mechanisms of the *Nox3* gene due to this very focal expression in the inner ear and functional data on *Nox3* have been only gathered in overexpression systems [36]. Most evidence regarding these mechanisms is derived from other isoforms, like *Nox2*, which is functionally similar to *Nox3* [29]. Thus, due to the limited literature, we relied on the other isoforms to formulate hypotheses about the mechanisms of susceptibility to NIHL.

Since ROS are commonly related to inflammation, an anti-inflammatory activity of NOX enzymes would seem illogical. However, over recent years there has been a striking number of publications pointing in the opposite direction. Most of the data about the anti-inflammatory activity of *Nox* enzymes comes from studies using mice deficient in the phagocyte NADPH oxidase *Nox2* as demonstrated by a decreased capacity to degrade phagocytized material in *Nox2*-deficient cells leading to the accumulation of debris [37]. Also, this hyperinflammation might be due to a lack of ROS-dependent signaling in *Nox2*-deficient phagocytes and ROS-dependent attenuation of Ca2+ signaling contributing to enhanced inflammation. Lastly, impairment of oxidative inactivation of proinflammatory mediators leads to a prolongation of the inflammatory response [30].

Hyperinflammation in NADPH oxidase-deficient mice was demonstrated in mouse models of Helicobacter gastritis [38][39], arthritis [40], demyelinating disease [41], and sunburn [42]. In experimental lung influenza infection, *Nox2* deficient mice demonstrated larger inflammatory infiltrates [43]. Also, by studying endothelial dysfunction, the absence of *Nox4* resulted in reduction of endothelial nitric oxide synthase expression, nitric oxide production, and heme oxygenase-1 expression, which was associated with apoptosis and inflammatory activation [44].

There is mounting evidence that NOX enzymes have a role in limiting the inflammatory response and we have shown this to be true in noise-induced cochlear damage. This anti-inflammatory activity of NOX enzymes is poorly understood in the cochlea. So far, as described in other isoforms, our initial hypothesis is that there are important protective mechanisms, such as an anti-inflammatory response resulting from noise exposure. This anti-inflammatory mechanism would be crucial to protect the cochlea against noise injury, overcoming its potential for damage caused by the release of ROS.

### Conclusion

In this manuscript we report the first functional validation of a gene of the auditory system arising from a GWAS. We demonstrate *Nox3* is involved in susceptibility to NIHL and mice deficient in *Nox3* are the most susceptible. This finding was specific to 8 kHz both physiologically (ABR threshold and DPOAE/ABR suprathreshold measures) and histologically at the level of the hair cell/auditory neuron synaptic ribbon. Our findings validate the power of the HMDP for detecting NIHL susceptibility genes and the tonotopic genetic susceptibilities present within the mouse cochlea.

## Supporting Information

S1 Fig GWAS results for 4 kHz post-noise exposure thresholds in the HMDP. Manhattan plot showing the association (-log10) p-values (-logP) for 4 kHz in 64 HMDP inbred mouse strains. The analysis was performed using 108,064 SNPs with a minor allele frequency > 5%. The level of significance at 4 kHz (rs33652818) is suggestive and does not reach genome-wide significance (p = 1.1x10^-4^).
(TIF)
