# Correction: Genome-Wide Association Study Identifies *Nox3* as a Critical Gene for Susceptibility to Noise-Induced Hearing Loss

**DOI:** 10.1371/journal.pgen.1005293

**Published:** 2015-06-11

**Authors:** Joel Lavinsky, Amanda L. Crow, Calvin Pan, Juemei Wang, Ksenia A. Aaron, Maria K. Ho, Qingzhong Li, Pehzman Salehide, Anthony Myint, Maya Monges-Hernadez, Eleazar Eskin, Hooman Allayee, Aldons J. Lusis, Rick A. Friedman

The affiliations for the twelfth and thirteenth authors are incorrect. Hooman Allayee is not affiliated with #4 but with #3 Department of Preventive Medicine and Institute for Genetic Medicine, USC Keck School of Medicine, University of Southern California, Los Angeles, California, United States of America.

Aldons J. Lusis is not affiliated with #3, instead he is affiliated with #4 Department of Human Genetics, University of California, Los Angeles, Los Angeles, California, United States of America and #6 Department of Microbiology, Immunology, and Molecular Genetics, University of California, Los Angeles, Los Angeles, California, United States of America.

In the Funding section, the California Institute for Regenerative Medicine grant (TG2-01161) is incorrectly assigned. This grant belongs to Amanda Crow, not to Hooman Allayee.

## References

[1] Lavinsky J, Crow AL, Pan C, Wang J, Aaron KA, Ho MK, et al (2015) Genome-Wide Association Study Identifies *Nox3* as a Critical Gene for Susceptibility to Noise-Induced Hearing Loss. PLoS Genet 11(4): e1005094 doi: 10.1371/journal.pgen.1005094 25880434 10.1371/journal.pgen.1005094 PMC4399881

