# Supplementary figures and images for: Genome-Wide Association Study Identifies Nox3 as a Critical Gene for Susceptibility to Noise-Induced Hearing Loss
Source: PLoS Genet. 2015 Apr 16;11(4):e1005094. doi: 10.1371/journal.pgen.1005094 (PMC4399881; doi:10.1371/journal.pgen.1005094)

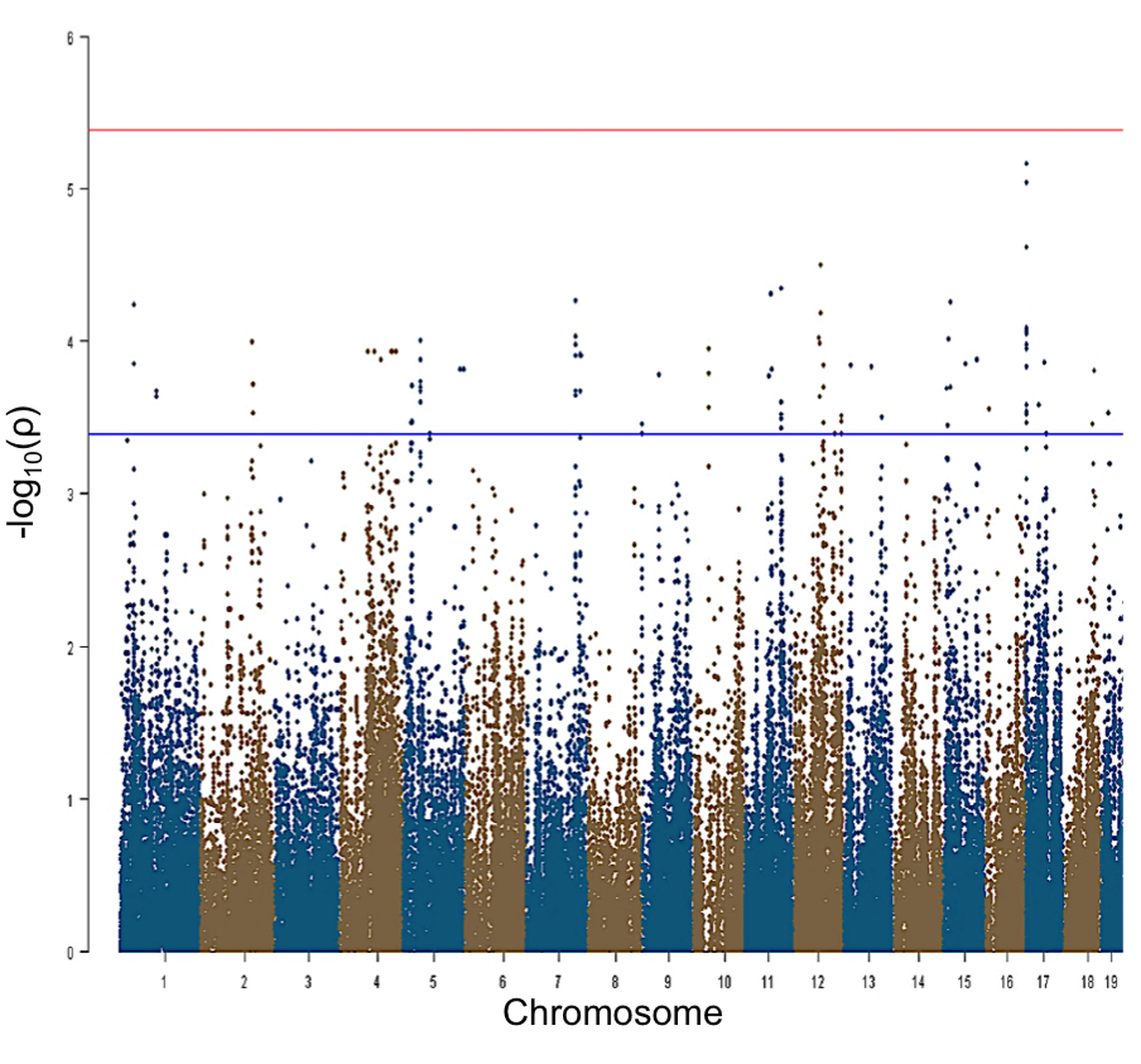

Supplement: S1 Fig — Manhattan plot showing the association (-log10) p-values (-logP) for 4 kHz in 64 HMDP inbred mouse strains. The analysis was performed using 108,064 SNPs with a minor allele frequency > 5%. The level of significance at 4 kHz (rs33652818) is suggestive and does not reach genome-wide significance (p = 1.1x10^-4^). (TIF) [file pgen.1005094.s001.tif]
